# Unraveling Binding Mechanism of Alzheimer’s Drug Rivastigmine Tartrate with Human Transferrin: Molecular Docking and Multi-Spectroscopic Approach towards Neurodegenerative Diseases

**DOI:** 10.3390/biom9090495

**Published:** 2019-09-17

**Authors:** Anas Shamsi, Taj Mohammad, Mohd Shahnawaz Khan, Moyad Shahwan, Fohad Mabood Husain, Md. Tabish Rehman, Md. Imtaiyaz Hassan, Faizan Ahmad, Asimul Islam

**Affiliations:** 1 Centre for Interdisciplinary Research in Basic Sciences, Jamia Millia Islamia, New Delhi 110025, India; 2 Department of Biochemistry, College of Sciences, King Saud University, Riyadh 11451, Saudi Arabia; 3 College of Pharmacy & Health sciences, Ajman University, Ajman, UAE; 4 Department of Food Science and Nutrition, Faculty of Food and Agricultural Sciences, King Saud University, Riyadh 11451, Saudi Arabia; 5 Department of Pharmacognosy, College of Pharmacy, King Saud University, Riyadh 11451, Saudi Arabia

**Keywords:** human transferrin, rivastigmine tartrate, spectroscopy, molecular docking, isotheral titration calorimetry, Alzheimer’s disease, neurodegenerative disorders

## Abstract

Studying drug–protein interactions has gained significant attention lately, and this is because the majority of drugs interact with proteins, thereby altering their structure and, moreover, their functionality. Rivastigmine tartrate (RT) is a drug that is in use for mild to moderate Alzheimer therapy. This study was targeted to characterize the interaction between human transferrin (hTf) and RT by employing spectroscopy, isothermal titration calorimetry (ITC), and molecular docking studies. Experimental results of fluorescence quenching of hTf induced by RT implied the formation of a static complex between hTf and RT. Further elucidation of the observed fluorescence data retorting Stern–Volmer and modified Stern–Volmer resulted in binding constants for hTf–RT complex of the order 10^4^ M^−1^ over the studied temperatures. Thermodynamic parameters of hTf–RT interaction were elucidated further by employing these obtained binding constant values. It was quite evident from obtained thermodynamic attributes that RT spontaneously binds to hTf with a postulated existence of hydrogen bonding or Van der Waals forces. Further, Circular dichroism spectroscopy (CD) also confirmed RT–hTf complex formation owing to upward movement of CD spectra in the presence of RT. ITC profiles advocated the existence of reaction to be spontaneous. Moreover, molecular docking further revealed that the important residues play a pivotal role in RT–hTf interaction. The findings of this study can be of a significant benefit to the drug-designing industry in this disease-prone era.

## 1. Introduction

The physiological functions of our body are governed by various factors where many essential elements play a vital role. Thus, maintaining proper levels of these elements is very important and the homeostasis is controlled through highly regulated mechanisms of uptake, storage, and secretion [1]. This disrupted homeostasis is implicated in many disorders ranging from Alzheimer’s (AD), Parkinson’s (PD), and Huntington’s (HD) diseases to amyotrophic lateral sclerosis (ALS) [2,3,4,5].

One of the important elements in this perspective is iron, which is amongst a vital trace element engaged in diversified physiological functions. The so-called “biometals” (iron, copper, or zinc) play a vital role in key metabolic processes and, hence, are considered life essential [6,7]. Free iron is a potent neurotoxin, and this toxicity can be owed to its redox activities. If homeostasis is disrupted, it might lead to cellular death or dysfunctionality [8]. Loss of iron may cause neurological disease, whereas deposition of free iron or its abnormal interaction with cellular components directly or indirectly contributes to neurodegenerative disorders [9]. Thus, it is quite evident that iron homeostasis disruption can be lethal in many ways. Therefore, maintaining iron homeostasis is a significant task, with human transferrin (hTf) and ferritin at the center. The intracellular pool of free iron, the labile iron pool (LIP), was well recognized and amends the expression of various proteins viz. amyloid precursor protein (APP) and many others [10,11].

There are various literatures that report the deposition of transition metals in nervous system in different neurodegenerative disorders, thereby advocating the role played by these metals [12,13]. Excessive iron deposition in the central nervous system (CNS) has been linked to neurodegenerative pathologies, namely AD, PD, ALS, and neuro-ferritinopathies and many more. Transferrin family is a group of proteins that function in the transport of iron around the blood stream after forming an iron–protein complex [14]. hTf is a glycoprotein which has a molecular weight of 79.6 kDa.

The pathological hallmarks of Alzheimer’s disease are β amyloids, which are insoluble deposits of 4 kDa peptides of ~40–42 amino acids and are key players in Alzheimer’s disease [15]. The importance of this disorder can take note from the fact that, globally, it affects nearly 40 million people coupled with a well-being budget of about $820 billion per year [16,17].

With developments in the pharmaceutical industry, thorough investigation of the interactions of important classes of therapeutics as well as of other potential drugs with either plasma or target tissue, proteins have been considered an important part of pharmacological profiling [18,19]. Studying drug–protein interactions have become imperative in this era where each new day a new disease is encountered and there are many existing lethal disorders against which drugs are being continuously tried and targeted. 

Rivastigmine tartrate (RT) is a carbamate inhibitor of acetylcholinesterase which is used for the treatment of mild to moderate Alzheimer’s disease in adults [20] and is approved by the US Food and Drug Administration [21]. Rivastigmine exhibits log linear pharmacokinetics at dosages up to 6 mg daily. The incidence rates of adverse events from clinical trials of rivastigmine appear less flattering than those of other acetylcholinesterase inhibitors. Outside the clinical trial setting, some clinicians may have found it difficult to escalate initial doses of rivastigmine to effective doses (6 to 12 mg) because of adverse effects. RT has been shown to improve patient’s performance in all the three major domains: cognitive function, global function, and behavior [22,23].

This study was intended to understand the interaction between RT and hTf and the elucidation of molecular mechanisms underlying this interaction. This study employed fluorescence spectroscopy observations coupled with molecular docking studies for better insight into the RT–hTf interaction. 

## 2. Materials and Methods

### 2.1. Materials

Human transferrin and Rivastigmine tartrate were purchased from Sigma-Aldrich Co. (St. Louis, MO, USA). Unless stated, all the chemicals were procured from Sigma. Double distilled and de-ionized water from a Milli-Q^®^ UF-Plus purification system (Merck, Kenilworth, NJ, USA) was used for preparation of all buffers.

### 2.2. Stock Solution of hTf and RT

Our experimentation involved preparation of a starting solution of 62-µM hTf. This was done in a 50-mM sodium phosphate buffer of pH 7.4. Correspondingly, a stock solution of 4-mM RT was made up in double distilled water. 

### 2.3. Steady State Fluorescence

Fluorescence spectroscopy was retorted for understanding the RT–hTf interaction. Our experimental parameters were as follows: quartz cuvette was used with a path length of 1 cm while the recording range was 300–400 nm with excitation performed at 280 nm. hTf (4 µM) was titrated with RT in a ratio of 1:8. This assay was carried out at three varying temperatures (301, 303, and 305 K). Further, Stern Volmer equation (Equation (1)) was deployed to evaluate reduced fluorescence intensity with a corresponding increase of the drug.

(1) $$\frac{F_{0}}{F}=1+K_{sv}\left[C\right]$$

where the highest fluorescence intensity of free hTf is depicted by *F*_0_, fluorescence intensity of the RT–hTf complex is shown by *F*, the Stern–Volmer constant is depicted by *K*_sv_, and [*C*] depicts the concentration of quencher (RT).

Static or dynamic quenching can occur for each process [24], and to have an insight into what sort of quenching is taking place in an RT–hTf interaction, Equation (2) was used.

(2) $$K_{q}=\frac{K_{sv}}{\tau_{0}}$$

where the apparent bimolecular quenching rate constant is *K*_q_; *K*_sv_ refers to the Stern–Volmer constant; and τ_0_ refers to the average integral fluorescence lifetime of tryptophan [25], which is ~5.78 × 10^−9^ s.

Further, using of double log relation, i.e., “Modified Stern–Volmer equation” (Equation (3)), binding sites (*n*) and binding constant (*K*) can be found.

(3) $$log\frac{F_{0}-F}{F}=logK+n\;\log\left[C\right]$$

where the highest fluorescence intensity of only hTf is *F*_0_ whilst *F* depicts fluorescence intensity in the existence of RT, *K* depicts the binding constant, *n* depicts the number of binding sites, and the concentration of RT is depicted by *C*.

Van’t Hoff’s equation (Equation (4)) [26] was used to calculate change in enthalpy and entropy at various temperatures. Each reaction is accompanied with change in energy, change in entropy, and change in enthalpy. Equation (5) was used to calculate free energy change involved in an RT–hTf interaction.

(4) $$\ln K=-\frac{\Delta H^{0}}{RT}+\frac{\Delta S^{0}}{R}$$

(5) $$\Delta G^{0}=\Delta H^{0}-T\Delta S^{0}$$

where *K* depicts the binding constant, ∆*H*^0^ refers to the enthalpy change while ∆*G*^0^ corresponds to the free Gibbs energy change, ∆*S*^0^ depicts the change in entropy, and R is the universal gas constant (1.987 cal mol^−1^ K^−1^).

### 2.4. Circular Dichroism Spectroscopy (CD)

CD spectra of free protein and drug protein were procured, making use of JASCO-J-1500 spectropolarimeter (JASCO INTERNATIONAL CO., LTD.11-10, Myojin-cho 1-chome, Hachioji, Tokyo 192-0046, Japan) connected with a Peltier-type temperature (PTC-517) controller along with USB 2.0/Spectra Manager™ or Spectra Manager™ CFR. The calibration of the instrument was done with d-10-camphorsulfonic acid. Our experimental parameters were as follows: recording range was 205–250 nm, constant temperature of 25 °C, scan rate of 100 nm/min with response time of 1 s, and 0.1 cm path length cuvette [26].

### 2.5. Isothermal Titration Calorimetry

ITC is an efficient technique for studying thermodynamic profiling of protein–drug interactions. To begin ITC, protein and ligands were thoroughly degassed for 30 minutes prior to loading of the samples in cells and syringes in a bid to remove any bubbles present in protein and ligand solutions. VP-ITC titration microcalorimeter (Micro Cal Inc., Northampton, MA, USA) coupled with Origin 8.0 software (Northampton, MA, USA, Origin Lab Corporation One Roundhouse Plaza Northampton, MA 01060 USA) was used to carry out ITC measurements at 25 °C. The reference cell was filled with double distilled water whilst the sample cell was filled with 20-µM hTf; 25 injections of degassed 1-mM RT solution were infused by rotating the syringe in the sample cell filled with the protein of interest. Each injection spanned 10 s, with 180 s being the spacing in two successive injections; the initial delay for the first one was 60 s, 307 rpm was the rotation speed of the syringe, and the reference power was 16 µcal s^−1^. 

### 2.6. Molecular Docking Analysis

This study was accomplished on DELL^®^ Workstation with Intel^®^ Xeon^®^ CPU E5-2609 v3 processor (Intel Corporation, Delhi, India), 64 GB RAM, and two TB hard disk (DELL Precision Tower 7810, DELL, New Delhi, INDIA) running on an Ubuntu 14.04.5 LTS operating system (Trusty Tahr, Canonical Ltd., London, UK). Online resources Protein Data Bank (PDB) [27] and PubChem [28] were used in the retrieval of the three-dimensional coordinates of hTf and RT. Bioinformatics tools AutoDock Vina [29], Discovery Studio [30] and PyMOL [31] were employed for docking and visualization purposes. 

Atomic coordinates of the hTf crystal structure were taken from PDB (ID: 3V83), and it was subsequently preprocessed in SPDBV [32] and AutoDock Tools [33]. Subsequently, co-crystalized ligands were removed from the native PDB coordinates file. RT was obtained from the PubChem database in the processed three-dimensional format. The docking was structurally blind for the compound where it was free to move and search the binding site(s) of the protein. Here, based on the binding affinity and scoring, the top binding pose among nine possible docked conformations of RT with hTf was selected.

## 3. Results and Discussion

### 3.1. Steady State Fluorescence Studies

**Figure 1:**
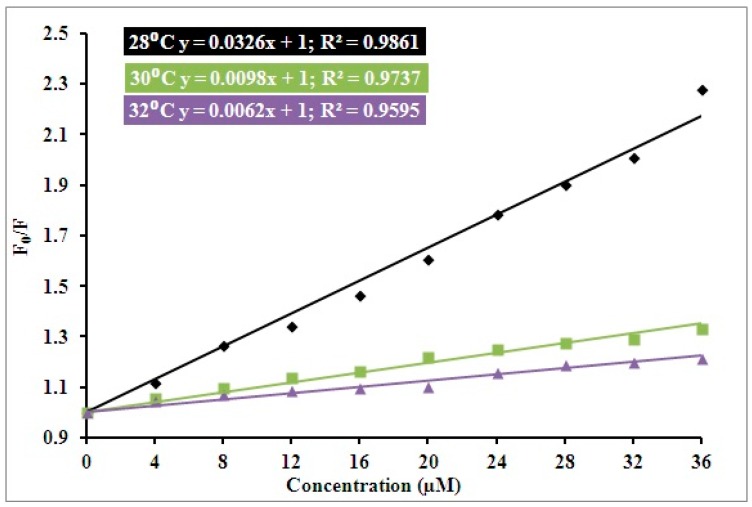
A plot of *F*_0_/*F* vs. [*C*] (Stern–Volmer plot) as a function of three different temperatures: The three temperatures in consideration are 301, 303, and 305 K.

When a complex is formed between proteins and ligands, intrinsic fluorescence can be retorted so as to be aware of this complex formation and further to find out various binding parameters for protein–ligand interaction. A concentration-dependent quenching of the intrinsic fluorescence of hTf by RT was observed upon spectral monitoring of the fluorescence intensity of the hTf–RT interaction (Figure 1). Fluorescence quenching refers to the event in which reduction in fluorescence intensity is observed with a corresponding increase in ligand. 

The binding parameters of the RT–hTf interaction were explored by performing intrinsic fluorescence assay at varying temperatures (301, 303, and 305 K). For native protein, there was a peak corresponding to 330 nm. However, there was an evident decrease in hTf fluorescence with increasing RT concentration, suggesting the formation of an hTf–RT complex (Figure 1). After recording fluorescence quenching, the next motto was to discover the operative mode of quenching for an hTf–RT interaction. 

Static quenching refers to the phenomenon in which a formed complex either acquires weak fluorescence or no fluorescence at all. On the contrary, dynamic quenching depicts the event in which fluorescent molecules and quenchers collide. Considerable quenching of a protein’s fluorescence intensity upon binding with ligands is typically either because of static formation of a non-fluorescent complex or dynamic molecular diffusion [34]. 

Temperature reliance of the quenching process can give an inner idea about the operative mode of quenching in an hTf–RT interaction. For this very reason, varying temperatures were taken into consideration for fluorescence quenching experiments.

Therefore, in a bid to differentiate between the two distinct mechanisms, the recorded spectral results were analyzed with the help of three well-known formulae viz. Stern–Volmer, Lineweaver–Burk, and the double-log relations [35,36].

In general, a hike in temperature would lead to elevated binding constant values for the dynamic type of quenching whilst a corresponding increase in binding constant with increasing temperatures would imply a static complex formation [37,38].

Equation (1) was deployed to calculate the values of *K*_sv_ (Stern–Volmer constant). The observed *K*_sv_ values of hTf–RT interaction can also be indicative of the form of quenching taking place between protein and ligand. A plot of *F*_0_/*F* vs. [*C*] depicted in Figure 1 gives the value of *K*_sv_. The slope of the plot of *F*_0_/*F* vs. concentration at a fixed intercept gives the value of *K*_sv_.

*K*_sv_ values at three varying temperatures are shown in Table 1. It is quite clear that with elevation in temperature, decreased *K*_sv_ values were observed and this type of variation in *K*_sv_ as a function of temperature indicates the existence of the static complex formation of RT and hTf. *K*_q_ (Quenching constant) value also gives an idea about the mode of quenching occurring for protein–drug interactions. The observed values of *K*_q_ as calculated by Equation (2) are in line with the obtained *K*_sv_ values (Table 1); *K*_q_ decreases with increasing temperature, thereby implying the mode of interaction as static for an hTf–RT interaction. 

**Figure 2:**
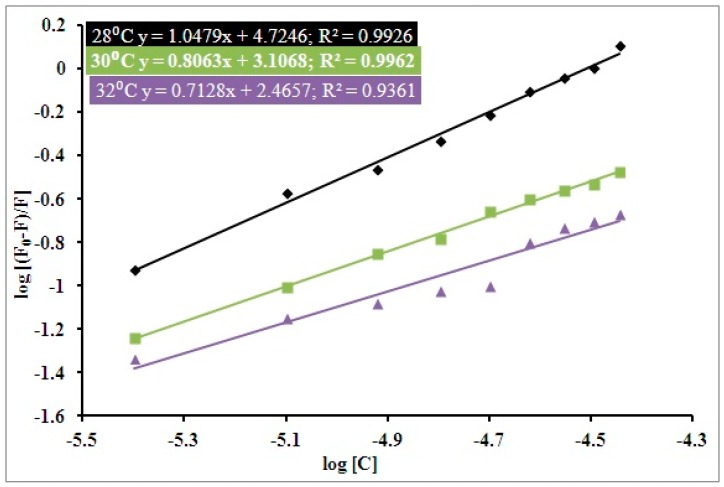
Double-log graphs (Modified Stern–Volmer plot) of the experimental fluorescence data for RT–hTf binding: The *x*-axis shows the log of concentration of quenchers while the *y*-axis depicts log *F*_0_ − *F*/*F*.

Further, Equation (3), the “Modified Stern–Volmer equation”, was deployed to have an understanding of hTf–RT interactions. A plot of Log [(*F*_0_ − *F*)/F] vs. log [*C*] in regard to the modified Stern–Volmer equation gives us the values of binding parameters for an hTf–RT interaction viz. binding constant (*K*) and number of binding sites (*n*). Figure 2 shows the plot of Log [(*F*_0_ − *F*)/*F*] vs. log [*C*]; the slope of this plot gives the number of binding sites (*n*). The intercept gives us the binding constant (*K*). The temperature dependency of the binding constant was taken into account as it gives confirmation about the effective mode of interaction for an hTf–RT interaction. It was quite evident that the binding constant (*K*) decreases with increasing temperature (Figure 2 and Table 2), thus implying a static mode of interaction for this process. Number of binding sites (*n*) was found close to unity at all three temperatures taken into consideration (Table 2).

### 3.2. Thermodynamic Features of hTf–RT Interaction

To further understand the mechanism involved in an hTf–RT interaction, thermodynamic characteristics of static complex formation were explored. Similarly, estimation of thermodynamic parameters was carried out viz. Gibbs free energy Δ*G*^0^, enthalpy (∆*H*^0^), and entropy (∆*S*^0^), employing Equations (4) (Van’t Hoff equation) and (5) which uses the gas constant *R*, the experimental temperature *T*, and the calculated *K* values in Table 2.

**Figure 3:**
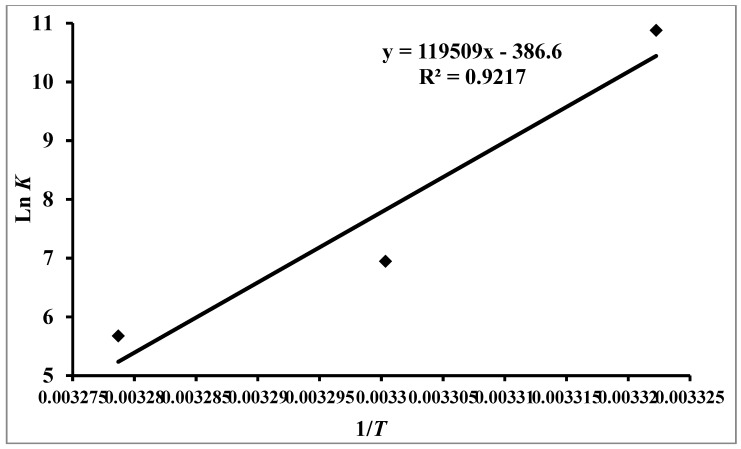
Van’t Hoff plot for the determination of thermodynamic parameters of RT–hTf interaction: The *x*-axis depicts the inverse of temperature in consideration while the *y*-axis shows natural log of the obtained binding constant obtained at these temperatures.

A graph having ln*K* on the *y*-axis against 1/*T* on the *x*-axis (Van’t Hoff plot) (Figure 3) produced a linear fit of the data points. The slope of the Van’t Hoff plot gives ∆*H*^0^whilst the intercept provides ∆*H*^0^. Subsequently, the values of ∆*G*^0^, ∆*H*^0^, and ∆*S*^0^ were calculated from the resulting linear regression equation and these values are depicted in Table 2.

There are many studies that have used these thermodynamic parameters to discover the potential binding forces between various ligands and proteins [39,40]. One of our previous studies reported binding between an important anticancer drug, temsirolimus, with a plasma protein, human serum albumin, thereby revealing the mechanism of interaction between these two [19].

**Figure 4:**
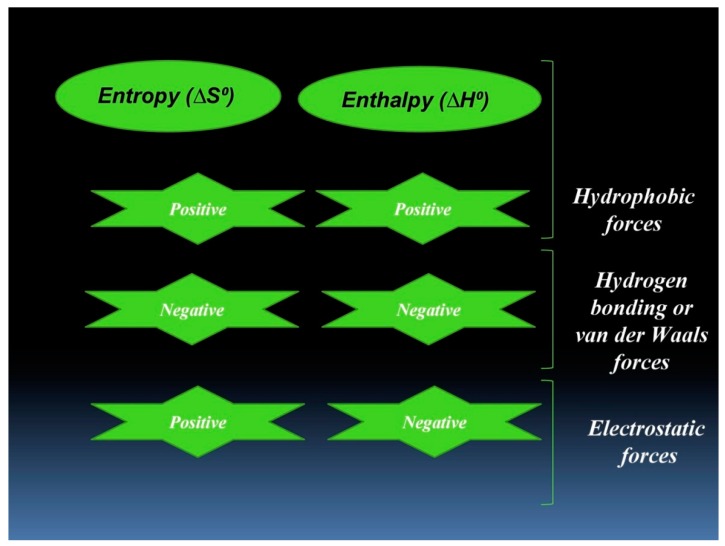
Graphical representation of the possible interaction forces depending upon thermodynamic parameters.

As reported in earlier studies, sign and magnitude of individual and/or combined values of entropy and enthalpy can be correlated to the prevailing noncovalent binding forces in a protein–ligand interaction as depicted in diagrammatic illustration (Figure 4). The results from this study and the values of entropy and enthalpy depicted in Table 2 advocate the existence of hydrogen bonding or Van der Waals-driven interactions that spontaneously take place between hTf and RT. Further, molecular docking provides a detailed investigation of the forces and residues playing a vital role in hTf–RT interactions. 

### 3.3. Circular Dichroism Spectroscopy

**Figure 5:**
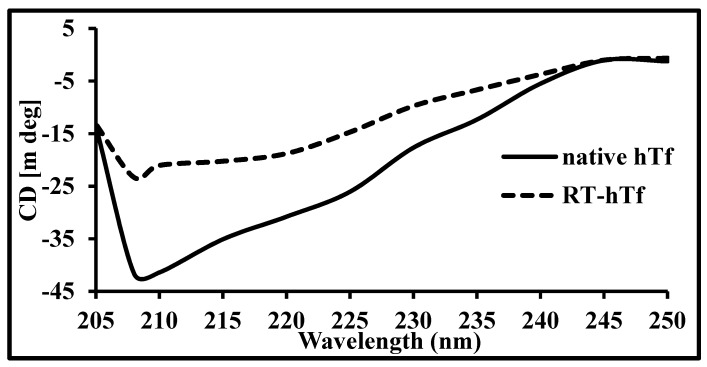
Far UV circular dichroism (CD) spectra of native hTf (—) and RT–hTf (1:8). The *x*-axis shows the wavelength range in which CD spectra is recorded while the *y*-axis shows CD in millidegrees.

One can analyze structural changes in proteins either due to interactions with ligands or some other cause by employing CD spectroscopy [41]. It is amongst imperative tools to scrutinize alterations in proteins once the ligand binds to a protein and alters its structure [42,43]. Figure 5 depicts the CD spectra of native hTf with a peak at around 208 nm which is characteristic of an alpha helix. Thus, this peak at around 208 nm clearly suggests that hTf is an alpha helix-rich protein. If the protein is alpha rich; it will show a peak at around 208 nm and 222 nm while beta sheet-rich proteins show a peak at around 218 nm [44].

An ascendant shift in the far UV CD spectrum indicates a reduction in helical structure. On the contrary, a descending progress in the spectrum suggests an augmented helical structure. Figure 5 shows the differences in CD spectra of free hTf and RT–hTf, clearly implying that RT induces structural changes in hTf: an upward movement clearly visible coupled with no significant peak shift. Thus, these observed changes in far UV CD spectra evidently imply that RT leads to structural loss of the alpha helix in native hTf [45].

### 3.4. Isothermal Titration Calorimetry (ITC)

**Figure 6:**
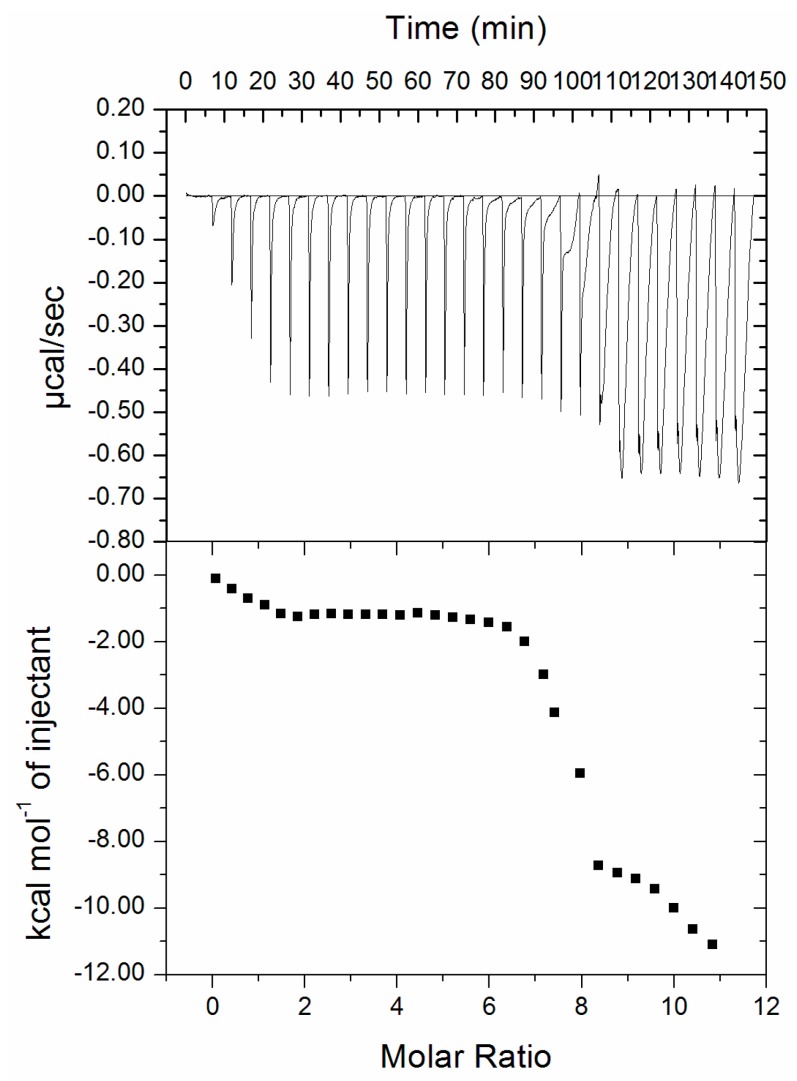
Isothermal titration calorimetry (ITC) profile of RT-hTf binding: Calorimetric responses owing to consecutive injections of RT in the ample cell with hTf are depicted in upper half whilst the lower panel shows integrated heats of interactions as a function of the [RT]/[hTf] molar ratio.

ITC is a routinely employed procedure to measure thermodynamic parameters of protein–ligand interactions [46]. The ITC profile of an RT-hTf interaction is depicted in Figure 6. It is quite evident from Figure 6 that an interaction takes place between RT and hTf in a spontaneous manner evident from the negative Gibbs free energy obtained for the RT–hTf interaction (Table not shown). The upper portion of the ITC profile corresponds to the raw data procured by consecutive injections of RT to hTf. Each heat burst curve depicts the amount of heat generated for each injection of the same amount of ligand. Origin 8.0 software is attached to VP ITC-200, and this is retorted for obtaining all graphs viz. ITC profile. A plot of heat vs. molar ratio is being shown in the bottom panel. ITC takes into account temperature while measuring thermodynamic parameters. In general, it is believed that there are variations in parameters obtained from fluorescence spectroscopy and ITC. It is a well-known fact that there are changes in thermodynamic parameters procured from ITC and fluorescence spectroscopy and that this is related to the fact that ITC measures a global change in the thermodynamic property whilst fluorescence spectroscopy takes into consideration only the local changes around the fluorophore (Trp-214) [47,48].

### 3.5. Molecular Docking Studies

**Figure 7:**
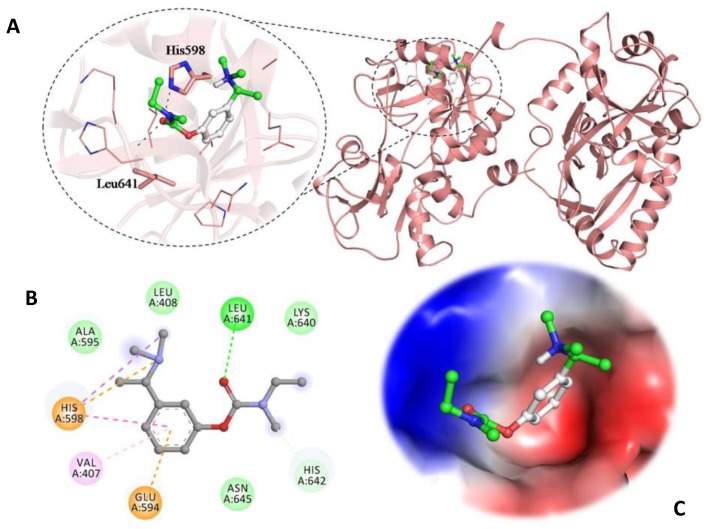
Cartoon representation of hTf in a complex with RT: Polar interactions sharing residues are shown in the stick element colour, and other interacting residues are shown in the line model. (**A**) Three-dimensional view of binding pocket residues of human serum transferrin interacting with RT. (**B**) Two-dimensional diagram of hTf residues interacting with RT. (**C**) Charged surface view of hTf binding pocket occupied by RT.

In order to understand the mechanism of binding between hTf and RT, we did molecular docking study. Here, we observed that the RT is showing significant binding affinity (−5.7 kcal/mol) towards hTf with several close interactions. There are many key residues of hTf that are at centre stage in hTf-RT interaction viz. His 598 and Leu 641. Several binding pockets were observed in the selected structure of hTf where RT can bind with different conformations. There are different binding poses where hTf is found to bind in the deep cavity of hTf more efficiently. Based on consensus binding affinity and significant interactions with the important residues, RT is being proposed as a possible binding partner of hTf. The selected bound conformation of RT with hTf is depicted in Figure 7. Many significant interactions viz. Hydrogen bonding (green), van der Waals (light green) and alkyl interactions (purple) are offered by the surrounding residues of hTf to RT and are being shown in Figure 7B. The analysis of its docked conformation with hTf revealed that the RT occupies the internal binding pockets with appreciable binding affinity and form several close interactions with the critically important residues. Figure 7C clearly depicts RT molecule in the binding pocket of hTf.

## 4. Conclusions

Insights into the molecular features of RT–hTf binding were gained by means of fluorescence and CD spectroscopy coupled with molecular docking studies. Fluorescence spectroscopy results implied that RT induced quenching of inherent fluorescence of hTf. Fluorescence experimentation was accomplished at three varying temperatures (301, 303, and 305 K) in a bid to find binding constant values (*K*) along with the number of binding sites (*n*). Mathematical calculations revealed that the observed quenching was due to static formation of the RT–hTf complex with a binding constant in the range of 10^4^ M^−1^, highlighting the potency of this interaction. The Van’t Hoff equation gave us the negative value of ∆*G*^0^, which implied the reaction was spontaneous and thermodynamically favorable. An upward movement of CD spectra in far UV CD of protein (hTf) in the presence of drug (RT) suggested that an hTf–RT complex is formed. The ITC profile of RT–hTf further gave confirmation of this interaction to be thermodynamically favorable and spontaneous. Structure-based molecular docking of RT with hTf was performed to see their interaction and bound conformations, and an appreciable binding between hTf and RT was observed. Molecular docking results clearly showed that RT occupies the internal binding pockets with appreciable binding energy and that it forms several close interactions with critically important residues.

In conclusion, this study demonstrates the binding mechanism of RT with hTf. This is first of its kind of study, where the mechanism of interaction between an important Alzheimer’s drug in use for Alzheimer therapy, RT, is delineated with a clinically relevant plasma protein, hTf. Understanding of the mechanism of interaction between RT and hTf shall provide an insight into various forces responsible for this interaction and details about the residues at heart of this drug–protein interaction. The findings of our study can be of significant benefit for Alzheimer therapy along with giving new prospects to the field of clinical medicine as it gives us a better understanding of binding mechanism of RT with hTf.

## Figures and Tables

**Table 1:** Thermodynamic parameters of Rivastigmine tartrate (RT)–human transferrin (hTf) system as calculated from fluorescence spectroscopy quenching experiments.

pH	Temperature (Kelvin)	*K*_sv_ (10^4^ M^−1^)	*K*_q_ (10^12^ M^−1^ s^−1^)	R^2^
**7.4**	301	1.1	1.90	0.88
	303	0.9	1.55	0.97
	305	0.6	1.03	0.95

**Table 2:** Thermodynamic parameters obtained for RT–hTf interaction as calculated from fluorescence spectroscopy quenching experiments.

pH	Temperature (K)	*K* (10^4^ M^−1^)	*N*	∆*G*^0^ (kcal mol^−1^)	∆*S*^0^ (cal mol^−1^ K^−1^)	∆*H*^0^ (kcal mol^−1^)	*T*Δ*S*^0^ (kcal mol^−1^)
7.4	301	5.3	0.99	−6.24451	−768.169	−237.465	−231.21
	303	0.10	0.99	−4.70917			−232.75
	305	0.02	0.93	−3.17283			−234.29

## Abbreviations

hTf	Human transferrin
RT	Rivastigmine tartrate
ITC	Isotheral titration calorimetry
CD	Circular dichroism
